# Problematic Substance Use among Sexual Minority and Heterosexual Young Adults during COVID-19

**DOI:** 10.3390/bs13080655

**Published:** 2023-08-04

**Authors:** Amanda K. Haik, Andrea M. Hussong

**Affiliations:** Department of Psychology and Neuroscience, University of North Carolina at Chapel Hill, Chapel Hill, NC 27599-3270, USA; hussong@unc.edu

**Keywords:** COVID-19 pandemic, substance use, young adult, sexual minority

## Abstract

Sexual minority young adults (SMYAs), compared to heterosexual young adults (HYAs), are a uniquely high-risk population for problematic substance use, a disparity perhaps exacerbated by the COVID-19 pandemic. This study tested whether SMYAs had more problematic substance use than HYAs during the pandemic due to isolation and loneliness as well as lower family closeness. Participants (*N* = 141) aged 23–29 completed self-report surveys in 2014–2015 as college students and in the summer of 2021 as young adults (59% White, 26% Black/African American, 9% Asian/Middle Eastern, 6% Hispanic/Latino, and <1% American Indian/Alaska Native). Results of multivariate regression and multiple group path analyses did not support hypothesized effects—SMYAs did not have greater increases in problematic substance use compared to HYAs, isolation and loneliness were not significant mediators, and family closeness was not a significant moderator. However, SMYAs experienced a lack of social safety—increased loneliness and decreased family closeness—compared to HYAs. Further research is needed to investigate both the impact and underlying processes of this decreased social safety on SMYA well-being beyond the pandemic to better inform tailored supports and interventions.

## 1. Introduction

Sexual minority young adults (SMYAs) are a high-risk population for problematic substance use [1,2] and are two times more likely to meet the criteria for a substance use disorder in the past 12 months compared to heterosexual young adults (HYAs) [2]. This disparity may be even greater during times of ecological stress, such as the COVID-19 pandemic [3]. In the current study, we drew on Minority Stress Theory to test how exacerbated stressors (e.g., isolation and loneliness due to pandemic-imposed restrictions) and depleted protective factors (e.g., compromised interpersonal support systems such as family closeness) may have disproportionately impacted problematic substance use among SMYAs as compared to HYAs during COVID-19. Continuing studies of how and why problematic substance use among SMYAs may have shifted during the pandemic are needed to inform pandemic recovery within this marginalized population, especially as long-lasting mental health concerns may persist beyond the acute phase of COVID-19 as with other major ecological stressors [3].

### 1.1. Minority Stress Theory and SMYA Pandemic Risk

A potential explanation for the increased risk of problematic substance use among SMYAs can be found in Minority Stress Theory [4]. Minority Stress Theory posits that two categories of stressors—minority stressors and general stressors—cumulate over the life course and lead to increased problematic substance use among SMYAs. Minority stressors refer to the unique and chronic stressors encountered by sexual minority individuals that link stigma due to one’s sexual identity with problematic substance use. Additionally, Minority Stress Theory refers to how adaptive coping mechanisms and social supports are protective factors decreasing the strength of the association between the cumulative effects of minority and general stressors and problematic substance use [4].

During COVID-19, pandemic control policies, such as social distancing and restriction of in-person gatherings, were experienced by many as a major life stressor leading to pervasive social isolation, loneliness, and compromised adaptive coping mechanisms [5,6]. Per Minority Stress Theory, the exacerbated stressors and depleted protective factors experienced during the pandemic may have increased problematic substance use more among SMYAs as compared to HYAs. Current pandemic-focused research has highlighted young adults as a uniquely high-risk population for isolation [7], loneliness [7,8], and elevated rates of anxiety and depressive symptoms [9], as well as a population at peak risk for substance use beyond the pandemic [10]. However, less attention has focused on pandemic-linked changes in young adult substance use, especially among marginalized young adult populations such as SMYAs [11]. Therefore, whether the disparity in problematic substance use between SMYAs and HYAs persisted or increased during the pandemic remains unclear.

Of the few studies addressing this question, the majority either did not use a HYA comparison group [12] or were cross-sectional [13]—making pandemic effects indiscernible. For example, in a cross-sectional study from the summer of 2020 (i.e., a few months after the first restrictions of the pandemic hit the US broadly), roughly 32% of SMYAs reported increasing their alcohol use when asked if their alcohol use had changed since the start of the pandemic [12]. This suggests that SMYAs perceived an increase in their substance use (or at least alcohol use) during the pandemic, but the study did not include a comparison group, nor did it address unique risk or protective factors for this population. Exploring the latter, a daily diary study of SMYAs with no pre-pandemic comparison found COVID news exposure (a unique pandemic stressor) did not predict daily alcohol and marijuana use but did predict higher coping motives for both substances [14]. Therefore, initial studies suggest complexity in understanding not only the unique vulnerability of SMYAs to problematic drinking during the pandemic but also possible risk and protective factors [11]. In the current study, we explored factors linked to using substances to cope with distress to understand how and why substance use disparities between SMYAs and HYAs may have persisted or exacerbated during the pandemic.

### 1.2. Impacts of Isolation and Loneliness before and during the COVID-19 Pandemic

COVID-19 was a unique opportunity to explore isolation (lack of supportive resources and interactions) and loneliness (the subjective feeling of lost close connections) as social distancing guidelines led to increases in both [6,7,15]. Both social isolation and loneliness have been linked to worse mental health and substance use outcomes in and out of the pandemic [7,15,16]. Furthermore, among young adults who reported increased feelings of loneliness, the majority also reported an increase in drinking and drug use during the pandemic [7]. Therefore, COVID-19-related isolation and loneliness were significant stressors that may have increased the likelihood of problematic substance use as a means to cope with resulting distress.

Though extant research demonstrates loneliness is particularly impactful to health disparities among SMYAs across age cohorts [17], few studies examine whether isolation and loneliness are particularly salient contributors to problematic substance use among SMYAs—a population with heightened vulnerability to substance use as a coping mechanism as indicated by Minority Stress Theory. A pre-pandemic meta-analysis by Gorczynski and Fasoli (2021) found only four published studies on loneliness comparing sexual minority and heterosexual individuals. Furthermore, only one of the four articles focused on the young adult age group, and it only explored mental health outcomes [18,19]. Therefore, no pre-pandemic study, to our knowledge, explored the effects of isolation and loneliness on problematic substance use comparing SMYAs and HYAs [18]. This gap persists in pandemic-focused research, even though SMYAs likely experienced greater isolation and loneliness during the pandemic than HYAs due to losses of sexual-minority-specific community supports and spaces (e.g., gay bars) as well as depleted protective factors such as connection to affirming peer and community support [20,21]. Therefore, the current study explored how COVID-19-related isolation and loneliness, likely through the mechanism of using substances to cope with distress, may have mediated increased rates of problematic substance use among SMYAs as compared to HYAs.

### 1.3. Impacts of Family Closeness before and during the COVID-19 Pandemic

Social support can often buffer the impact of stress on problematic substance use by alleviating resulting distress, serving as a source of resilience by promoting adaptive coping [4,22,23,24]. Pre-pandemic research shows that family support is a particularly impactful form of social support among SMYAs, with meta-analysis results finding that parental support is negatively correlated with substance use among SMYAs [25]. Furthermore, in results from the Add Health study, which is a prospective study following youth from adolescence through young adulthood, same-sex attraction in youth was associated with lower quality parent-child connection, which in turn was associated with problematic alcohol use in young adulthood [26]. Thus, family closeness, which is an indicator of support, may buffer problematic substance use among SMYAs by increasing feelings of social support and the use of adaptive coping resources. Alternatively, a lack of closeness may heighten problematic substance use among SMYAs as an additional source of minority stress, particularly if the lack of closeness is linked to family rejection of sexual identity.

During the COVID-19 pandemic, the structure and nature of family interactions likely changed for many young adults due to social distancing guidelines and other COVID-related stressors, resulting in less in-person access to family support for some [5]. Given its unique role in buffering minority stress, reductions in family support may have impacted SMYAs more than HYAs [25,26]. Extant research supports this hypothesis [27]. However, these pandemic-related studies only investigated the main effect of family relationships on mental health outcomes [28]. In the current study, we explored how family closeness served as a moderator of stress-related drinking (linked to isolation and loneliness) during the pandemic. This may occur in two ways. First, Minority Stress Theory suggests that social support (i.e., family closeness) is a protective factor decreasing levels of general and minority stressors among SMYAs and the overall levels of distress with which they must cope. Second, family closeness alleviates distress associated with loneliness and isolation, reducing the use of substances to cope. No studies to our knowledge have explored the role of family closeness as a buffer in stress-related substance use among SMYAs and HYAs during the pandemic.

### 1.4. Current Study

The current study addressed whether disparities in problematic substance use between SMYAs and HYAs expanded during the COVID-19 pandemic. Following Minority Stress Theory, we explored how COVID-19-related isolation, loneliness, and family closeness may be part of the underlying mechanism of using substances to cope with distress, leading to potentially increased problematic substance use during the pandemic. Specifically, we tested the following three hypotheses using longitudinal data collected before and during the pandemic: (1) SMYAs will have greater increased problematic substance use compared to HYAs during the pandemic; (2) SMYAs will have greater isolation and loneliness than HYAs during the pandemic which will partially account for increased problematic substance use; and (3) greater family closeness will reduce SMYA risk for isolation and loneliness and stress-related problematic substance use. Better understanding stress-based problematic substance use through this constellation of factors is critical for guiding healing within the SMYA community post-pandemic.

## 2. Methods

### 2.1. Sample

Participants were a subset of 840 alcohol-exposed college students (aged 18–23; M = 19.8) assessed mostly in 2015 (a few assessments in 2014) as part of a larger study (Time 1, “T1”) [29]. Those who also successfully completed a short-term follow-up study two weeks later regarding text messaging (*n* = 267) [30] were invited to complete an additional online follow-up survey about six years later in the summer of 2021 (now aged 23–29; M = 26.0) (Time 2, “T2”). A total of 142 participants completed the pandemic follow-up survey, with attrition due to incomplete survey (*n* = 24), refusing to participate (*n* = 7), unresponsive to contact attempts (*n* = 60), or unlocatable (*n* = 34). Analyses included 141 young adults (*n* = 1 dropped for missing sexual orientation data). Participants had a mean age of 26 and were 20% sexual minority, 61% women, 41% multiethnic, 85% employed, and 60% lived in their own residences (see Table 1).

### 2.2. Procedure

At T1, participants completed consent procedures and one of two randomly assigned computer-administered versions of the same survey during two in-person 75–90-min testing sessions separated by two weeks. They were compensated $20 and $25, respectively. At T2, participants completed an online consent procedure and a 45-min survey administered remotely via Qualtrics and were compensated with a $30 gift card. All procedures were approved by The University of North Carolina at Chapel Hill Institutional Review Broad.

### 2.3. Measures

#### 2.3.1. Sociodemographic Variables

Participants reported their age at T1 as well as their race/ethnicity, employment status [31], residential status, and gender at T2. Participants were asked to indicate if they were Hispanic or Latino and to indicate their racial identity by selecting all applicable response options [32]. For analyses, participants’ race/ethnicity was recategorized as non-Latino and White (coded as 0) or as a racial/ethnic Minority group (coded as 1). Employment was recategorized as unemployed or volunteer (coded as 0) or employed (coded as 1). Residential status was recategorized as living in one’s own residence (coded as 0) or in a shared or family residence (coded as 1). Because all gender-diverse individuals also identified as having a sexual minority identity, gender was not used in analyses but was measured as a descriptive statistic for the sample [33]. We assessed sexual orientation (only at T2) by asking, “Which of the following best represents how you think of yourself?” [33,34]. Due to the limited sample size, we recategorized sexual orientation as heterosexual young adults (“HYAs”; *n* = 113; combines straight and undefined/unsure/questioning sexuality; coded 0) and sexual minority young adults (“SMYAs”; *n* = 28; combines gay, lesbian, bisexual, queer, pansexual, and asexual; coded 1). Within the HYA group, we conducted *t*-tests across the primary outcomes to determine whether there were any systematic differences between the straight (*n* = 98) and undefined sexuality (*n* = 15) individuals that would make combining them problematic. No significant differences were found between these groups.

#### 2.3.2. Isolation

Participants reported isolation experiences due to COVID-19 at T2 with a subset of 7 items from The Epidemic–Pandemic Impacts Inventory (EPII) measure [35]. This subset of items assessed physical isolation or separation from social networks during the pandemic (coded as 0 = No, 1 = Yes). Items were selected from 14 face-valid items subjected to an exploratory factor analysis that supported a two-factor solution. Three items tapping disease-related isolation (e.g., quarantined due to disease; M = 0.36, SD = 0.36, Cronbach’s α = 0.63) and four tapping social network-related isolation (e.g., separation from friends and family, canceled social events, and inability to participate in social activities; M = 0.78, SD = 0.26, Cronbach’s α = 0.55) were separately averaged to form two isolation variables for analysis. As has been argued for stressful life events measures of other kinds, low-reliability estimates may not pose a particular problem because these types of measures are not governed by an underlying latent factor. Therefore, in our measure, the items are predictive of isolation rather than caused by isolation (as in effect- rather than causal-indicator models), which helps explain the low reliability estimates [36].

#### 2.3.3. Loneliness

Participants reported loneliness in the past month at T2 with the 20-item UCLA Loneliness measure, which has shown strong reliability and validity in prior studies [37]. Participants reported the extent to which the items described themselves on a 4-point scale from 1 (I never feel this way) to 4 (I often feel this way). Scores were calculated by averaging across items, with higher scores reflecting higher levels of loneliness (M = 1.91, SD = 0.68, Cronbach’s α = 0.95).

#### 2.3.4. Family Closeness

Participants reported perceived family closeness at T2 with 12 items for each parent assessed separately from a measure adapted by Barrera et al. (1993) [38] from the Network of Relationships Inventory [39]. The subscale included items assessing companionship, intimate disclosure, emotional support, approval, and satisfaction. Participants reported the frequency with which they experienced each of the items on a 5-point scale from 0 (Little or none) to 4 (The most). Scores for the subscale were calculated by averaging ratings across the combined mother and father figure items, with higher scores reflecting greater closeness (M = 1.56, SD = 0.73, Cronbach’s α = 0.94). To assess the moderating role of family closeness in the multiple-group analysis, we created a family closeness grouping variable coded low (below the median and coded 0; *n* = 65) or high (i.e., above the median and coded 1; *n* = 66).

#### 2.3.5. Problematic Alcohol and Drug Use

Participants completed 11 items from the Monitoring the Future (MTF) survey—a large, nationally representative dataset monitoring prevalence and patterns of young adult substance use in the United States for the past nearly 50 years—assessing heavy drinking and substance use [40]. Participants reported heavy drinking (i.e., 5 or more drinks in a row) at T1 and T2 by indicating their frequency of heavy drinking in the past year on a 7-point scale from 0 (0 occasions) to 6 (40 or more) (T1: M = 2.36, SD = 1.89; T2: M = 2.31, SD = 2.01). Participants reported an average number of drinks on any one occasion at T1 and T2 with a write-in response. For responses that included a range rather than a single number (e.g., 3–4 drinks), we used the maximum amount listed. With winsorizing, we capped responses at the 99th percentile, which excluded *n* = 1 (T1: M = 3.64, SD = 1.92; T2: M = 2.67, SD = 1.72). Participants reported past year drug use at T1 and T2 with items assessing 1) frequency of cannabis use (i.e., one item for weed, pot, hash, and hash oil), 2) frequency of cigarette use (i.e., maximum reported frequencies on four separate items for cigarettes, smokeless tobacco, cigars/pipe tobacco, and electronic cigarettes), 3) frequency of hallucinogen use (i.e., one item for LSD, MDMA, and other hallucinogens), and 4) frequency of stimulant use (i.e., maximum reported frequencies on three separate items for cocaine, amphetamines, and Adderall (not as prescribed)). Participants reported the frequency of drug use in the past year for each item on a 7-point scale from 0 (0 occasions) to 6 (40 or more). We retained the original response scale in variables for cannabis use (T1: M = 1.83, SD = 2.09; T2: M = 1.99, SD = 2.36) and cigarette use (T1: M = 1.01, SD = 1.64; T2: M = 0.96, SD = 1.81) because they met distributional assumptions for skew (i.e., below two) and kurtosis (i.e., below seven) for using confirmatory factor analyses and multivariate regression models [41,42]. Due to low base rates and violations of normality in the distributions, we collapsed hallucinogen and stimulant use by using the maximum reported frequencies, creating a dichotomized variable indexing “other drug use” (0 = no use, 1 = 1+ use) (T1: M = 0.25, SD = 0.43; T2: M = 0.25, SD = 0.43).

Participants also completed the Rutgers Alcohol Problem Index (RAPI) to assess alcohol problems. This unidimensional instrument has shown strong internal consistency in prior studies (Cronbach’s α = 0.92) [43] and good to excellent test-retest reliability at 1-month, 6-months, and 1-year testing intervals (r = 0.89–0.92) [44]. Participants reported the number of times they experienced a negative consequence related to their alcohol use in the past year on a 4-point scale from 0 (None) to 3 (More than 5 times). Participants only indicated whether they experienced the problem in the past year if they previously indicated experiencing the negative consequence in their lifetime. Scores were calculated by averaging ratings across the 23 items, with higher scores reflecting more endorsement of alcohol problems (T1: M = 0.21, SD = 0.29, Cronbach’s α = 0.74; T2: M = 0.13, SD = 0.22, Cronbach’s α = 0.87).

## 3. Results

### 3.1. Preliminary Analyses

There is no single analysis to determine power for the most complex model in this study (i.e., hypothesis 3). Therefore, we chose to calculate power for the multiple group comparison tests, which focus on the moderated mediation hypothesis. This statistical test is a chi-square difference test with six degrees of freedom (i.e., the number of degrees of freedom differentiating the covariate-only constrained multiple group model and the covariate plus mediational pathway constrained model). To test this model, we looked at power for a chi-square test with six degrees of freedom and a non-centrality parameter of 0.1 with a sample size of 141. Results showed that we had power of at least 0.80 to detect small to medium (effect size) group differences in at least one of the mediational pathways (given that the null holds). This suggests that this model is adequately powered to correctly reject the null hypothesis that SMYAs and HYAs differ in any of these mediational pathways. Furthermore, since this is the most complex model in the study, there is adequate power for all other proposed models as well.

Multiple imputation was used to account for missing data (with 100 imputations), as estimated in Mplus 8.0 [45]. Fit indices assessing model fit included the comparative fit index (CFI) and Tucker–Lewis index (TLI; with values >0.90 indicating acceptability) and the root mean square error of approximation (RMSEA; with values <0.08 indicating acceptability) [46]. We checked assumptions for each model, including tests of linearity (for continuous outcomes), homoscedasticity, outliers, and residual normality.

We began by creating four factor-score regression composite indices for problematic alcohol use and problematic drug use at T1 and T2 using confirmatory factor analyses conducted in Mplus 8.0 [45]. Problematic alcohol use scores were created from three indicators: heavy drinking, average number of drinks, and alcohol problems. Problematic drug use scores were created from three indicators: cannabis use, cigarette use, and other drug use. Modification indices suggested local dependence between the indicators of cannabis use and other drug use accounted for poor model fit, and when this covariance was added to the two measurement models, fit indices were all acceptable to strong for both T1 (χ^2^(7) = 7.45, *p* = 0.38; RMSEA = 0.02, CFI = 1.0, TLI = 0.99) and T2 (χ^2^(7) = 7.54, *p* = 0.37; RMSEA = 0.02, CFI = 1.0, TLI = 0.99). All items loaded significantly on each factor (*p* < 0.01) in each model. Then, we extracted factor score regression composites for the two-factor solution of problematic alcohol use and problematic drug use at T1 and T2. Means, standard deviations, estimates of internal reliability, and bivariate correlations among continuous study variables are presented in Table 2.

Differences between SMYAs and HYAs groups in study predictors and outcome variables were assessed using *t*-tests. Differences across sexual orientation were found such that SMYAs, compared to HYAs, had higher levels of loneliness (t(129) = −2.59; *p* < 0.05) and lower levels of family closeness (t(129) = 2.58; *p* < 0.05). No differences were found in disease-related isolation, social network-related isolation, T1 and T2 problematic alcohol use, and T1 and T2 problematic drug use. Paired-sample *t*-tests were conducted to compare substance use indicators (i.e., heavy drinking, average number of drinks, alcohol problems, cannabis use, cigarette use, and other drug use) at T1 and T2. Differences in substance use indicators were found across time points, suggesting higher rates of general substance use at T1 compared to T2. Specifically, there were significant differences in the average number of drinks at T1 (M = 3.64, SD = 1.92) and T2 (M = 2.67, SD = 1.72); t(135) = 5.50, *p* < 0.001; and alcohol problems at T1 (M = 0.21, SD = 0.29) and T2 (M = 0.13, SD = 0.22); t(140) = 2.95, *p* < 0.01.

### 3.2. Primary Analyses

We first conducted a multivariate regression analysis to establish covariates for analyses testing study hypotheses predicting problematic alcohol and drug use from participant age, race/ethnicity, employment status, and residential status (see Table 3, Model 1). Older participants had less T2 problematic alcohol use (b = −0.12, t = −2.50, *p* < 0.05) and T2 problematic drug use (b = −0.11, t = −2.42, *p* < 0.05) than younger ones. Additionally, participants with greater T1 problematic alcohol use had greater T2 problematic alcohol use (b = 0.81, t = 3.14, *p* < 0.01), and participants with greater T1 problematic drug use had greater T2 problematic drug use (b = 1.02, t = 3.98, *p* < 0.001). No other covariates served as significant predictors. Therefore, covariates for primary analyses included only age and T1 indicators of problematic alcohol and drug use, respectively.

To test hypothesis 1, we conducted a single multivariate regression by regressing T2 problematic alcohol use (using a linear link function) and T2 problematic drug use (using a logistic link function) on sexual orientation, controlling for age and the respective T1 problematic use indicator (see Table 3, Model 2). Sexual orientation was not significantly related to changes in T2 problematic alcohol use (b = 0.15, t = 0.95, *p* = 0.34) or T2 problematic drug use (b = 0.03, t = 0.19, *p* = 0.85).

**Figure 1:**
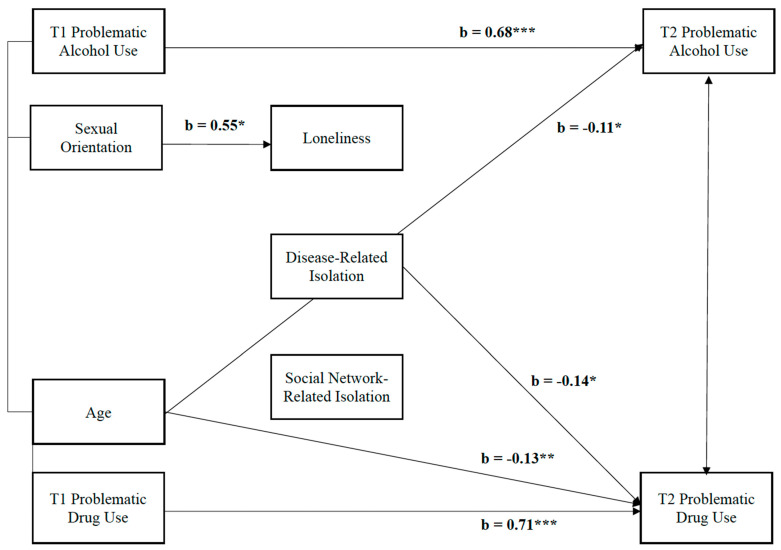
Full mediational path analysis: Isolation and loneliness as mediators between sexual orientation and problematic substance use. Note. All pathways are estimated. Only pathways significant at *p* < 0.05 are depicted. * *p* < 0.05; ** *p* < 0.01; *** *p* < 0.001.

To test hypothesis 2, we estimated a single path analysis by adding disease-related isolation, social network-related isolation, and loneliness as mediators to the multivariate regression model for hypothesis 1 (see Figure 1). Although sexual orientation was not related (directly or indirectly) to either problematic substance use outcomes, SMYAs had increased loneliness as compared to HYAs (b = 0.55, t = 2.74, *p* = 0.01). In addition, participants with greater disease-related isolation did show less T2 problematic drug use (b = −0.14, t = −2.16, *p* < 0.05). No other pathway was significant.

To test hypothesis 3, we conducted a multiple-group path analysis to test whether the mediational pathway (estimated in hypothesis 2) differed for those with high versus low family closeness. First, we used χ^2^ difference tests to evaluate whether an unconstrained model (in which no pathways were held equal across the two groups) fit the data significantly better than a model in which covariate pathways (i.e., age and the respective T1 outcome) were constrained to be equal across groups. We estimated this model separately for T2 problematic alcohol and drug use and found no differences between the covariate-constrained and unconstrained models (T2 problematic alcohol use: χ^2^(2) = 0.11, *p* = 0.95; T2 problematic drug use: χ^2^(2) = 0.47, *p* = 0.79). We then conducted χ^2^ difference tests for each outcome to evaluate whether the model with only covariate-constrained pathways fit the data significantly better than a model with all pathways in the mediational model constrained to be equal across groups. No differences were found between the covariate-constrained and fully-constrained models (T2 problematic alcohol use: χ^2^(6) = 3.34, *p* = 0.77; T2 problematic drug use: χ^2^(6) = 4.32, *p* = 0.63). These results indicate that in this sample, which is noted to be small and minimally representative, family closeness does not moderate the mediational pathway tested in hypothesis 2.

## 4. Discussion

Although Minority Stress Theory suggests that exacerbated stressors (e.g., isolation and loneliness due to pandemic-imposed restrictions) and depleted protective factors (e.g., compromised interpersonal support systems such as reduced family closeness) during COVID-19 may have disproportionately increased problematic substance use among SMYAs as compared to HYAs, we found no such differences in the current study. However, we did find that SMYAs had greater loneliness, though not isolation, than HYAs during the pandemic. We also found that isolation related to illness (of oneself or loved ones), though not loneliness or social network-related isolation, predicted decreases in problematic drug use during the pandemic. Although family closeness was lower in SMYAs than HYAs, stress-related substance use was not buffered by high closeness nor exacerbated by low closeness during the pandemic. Given the lack of studies focused on factors impacting patterns of problematic substance use during the pandemic for SMYAs versus HYAs, we consider each of these findings in turn.

### 4.1. SMYA Well-Being during the Pandemic

COVID-19-specific research has found mixed results regarding substance use disparities between SMYAs and HYAs [12,13]. The current study aligned with results demonstrating no group difference in problematic substance use between SMYAs and HYAs during COVID-19. We offer two interpretations of this null finding. First, our study was conducted during the first year of the pandemic, and thus this result should be interpreted as “developing”, as the association between the pandemic mental health crisis and substance use has yet to fully unfold, especially among marginalized populations [11].

Second, in the current study, we compared problematic substance use during the pandemic with use six years prior—a developmental period in which normative declines in substance use are associated with maturing out of use—and found little change in any indicator of substance use. Therefore, we cannot fully disentangle whether there was an acute increase or decrease in problematic substance use in our sample with the pandemic, only that whatever between group changes occurred across this six-year period were fairly parallel across HYAs and SMYAs. Among the broader young adult population, evidence suggests that substance use decreased during the beginning of the pandemic before rebounding in 2021 [11,47]. Though this trend is tentative and qualified based on individual and contextual differences, it aligns with the idea that pandemic-related changes in substance use may be more related to decreased access (and thus decreased substance use) rather than with increased stress (and thus increased substance use), at least early in the pandemic. Pandemic control policies may have particularly restricted access for SMYAs with the loss of queer spaces (e.g., gay bars), which were often drinking-oriented spaces [48,49]. As a result, substance use may have decreased more among SMYAs due to the unique lack of access and socialization opportunities to queer spaces, lessening the disparity between SMYAs and HYAs. Differences in the reasons for possible changes in substance use between these two groups require more investigation.

Even though we did not find disparities in problematic substance use, we did find differences between SMYAs and HYAs in stress indicators during the pandemic (i.e., isolation and loneliness). More specifically, SMYAs experienced increased loneliness, but not isolation, as compared to HYAs during the COVID-19 pandemic. This finding was consistent with pre-pandemic research showing that SMYAs generally experience heightened levels of loneliness as compared to HYAs [19]. Yet the source of this loneliness (subjective feeling of lost close connections) does not appear with differences in isolation (lack of supportive resources and interactions) [15]. Both perceived disease-related (e.g., quarantined due to disease exposure) and social network-related (e.g., separation from friends/family and social activities) isolation were similar across HYAs and SMYAs. Thus, isolation was a more general experience among young adults during the pandemic, whereas loneliness was more salient in SMYAs.

This may have occurred because SMYAs experienced the loss of sexual minority-specific peer and community support during the pandemic, limiting connection with “safe” spaces, both physical and relational. Therefore, SMYAs may have experienced an exacerbated lack of social safety (i.e., reliable connection, inclusion, and protection in social relationships), which may have led to increased levels of loneliness as compared to HYAs [50]. Future work should continue to not only tease out the pervasive effects of loneliness on SMYA psychosocial well-being but also explore ways of facilitating and increasing connection among this population vulnerable to disconnection, especially during times of heightened ecological stress, specifically by exploring sources and types of social support to increase social safety among this unique population.

Counter to expectations; we found that participants with greater disease-related isolation, but not social network-related isolation or loneliness, had less T2 problematic drug use. This result stands in contrast to a self-medication mechanism as explaining pandemic-linked changes in substance use and instead aligns with the explanation that changes in access, including restrictions to both substances themselves and the spaces to use them socially, resulted in lower problematic substance use early in the pandemic [11,51].

### 4.2. The Role of Family Closeness

Although family closeness did not moderate stress-related substance use among SMYAs during the pandemic, family closeness did play another role. SMYAs reported lower family closeness as compared to HYAs during the COVID-19 pandemic, consistent with research in and out of the pandemic [26,28]. Other studies showed that for some young adults, pandemic controls decreased engagement in typical social and extracurricular contexts and changed structures of social networks, possibly leading to more interactions with family [5]. These interactions with family may have differentially impacted levels of stress for different groups of young adults. For SMYAs, prior pandemic-focused research suggested the family context may have heightened stress levels as these individuals were forced to isolate or engage with unsupportive families (i.e., homophobic parents) [20]. In doing so, it is possible these experiences resulted in lower perceptions of family closeness among SMYAs as compared to HYAs.

However, even though we found SMYAs had lower family closeness as compared to HYAs, lower family closeness did not pose any unique risk of greater isolation and loneliness as compared to SMYAs with higher family closeness. Perhaps the reason why family closeness did not differentially impact the risk for isolation and loneliness in SMYAs as compared to HYAs is that there are more impactful sources of social support besides family (e.g., peers, sexual minority community groups). For example, Parra et al. (2018) found peer social support buffered the link between poor family support and internalizing symptoms [52]. Thus, it may be that sources of support other than family are particularly salient to buffer SMYA stress experiences. Investigating how these different sources of social support are perceived by SMYAs is key to understanding how social support is best received, as it is not just “any” support but “desired” support that may increase feelings of social safety in this population [50]. Furthermore, family support may not be a particularly salient form of social support to this age group. Young adults are exploring newfound independence and identity formation, thereby potentially distancing themselves generally from family relationships [53,54]. In our sample, only 10% lived in the residence of a family member. Therefore, though family closeness was found to be lower among SMYAs compared to HYAs, it did not impact experiences of isolation and loneliness, suggesting the need to investigate other, more meaningful forms of social support for this young adult population holding a unique minority identity.

### 4.3. Limitations

Despite its strengths, the current study also has limitations. First, though a power analysis revealed sufficient power for our most complex analysis, the SMYA sample was small, limiting external validity and exploration of heterogeneity within this group. Prior research suggests that some SMYA subgroups experience more severe substance use symptoms than others outside of the pandemic (e.g., including bisexual individuals and those who hold additional marginalized gender or racial/ethnic identities) [55,56,57]. Future work is needed to understand heterogeneity within SMYAs for substance use during the pandemic. Second, the six-year gap between T1 and T2 problematic substance use assessment confounds any potential pandemic effects with those due to maturation and other ecological stressors. Therefore, the results of this study are relevant during COVID-19; however, they cannot be interpreted as due to pandemic effects. This remains to be seen. Third, participants filled out some measures only at T2 (i.e., pandemic-related loneliness and isolation, as well as family closeness). Accordingly, we do not have baseline measurements of these variables as comparison points.

The strengths of the study lie in the novelty of the question. We are one of the first studies to explore problematic substance use as a primary outcome with this specific constellation of variables associated with problematic substance use that are both exacerbated stressors and depleted protective factors during the pandemic, particularly for SMYAs. Additionally, this study was a between-group comparison between SMYAs and HYAs, and many studies do not include HYAs as a comparison group.

## 5. Conclusions

Overall, the current study was the first to our knowledge to explore how and why specific stressors impact the potentially exacerbated problematic substance use disparity between SMYAs and HYAs during the COVID-19 era. Novel findings highlighted how SMYAs did not experience heightened levels of problematic substance use during the pandemic; they experienced a lack of social safety (e.g., increased loneliness and decreased family closeness). This finding is tentative and qualified based on design limitations (i.e., family closeness and loneliness were only assessed at T2) that do not allow us to disentangle whether this lack of social safety was unique to the pandemic or not. Nonetheless, the impacts of this lack of social safety on mental health problems can extend years into the future [58]. Long-term negative health outcomes of loneliness can include prolonged activation of the sympathetic nervous system due to increased vulnerability to and intensity of external stressors, decreased cardiovascular health, and other physiological deficits (e.g., slower wound healing and poorer sleep efficiency) [59,60,61]. Therefore, as the yet-to-be-seen pandemic ripple effects continue to unfold, it is important to understand how to support SMYAs with points of connection that address this lack of social safety. This is important as existing literature shows having a sexual minority identity was significantly correlated with expressing a need for and experiencing barriers to care during the pandemic [62]. Thus, focusing research on supporting clinical efforts for this uniquely vulnerable population during the pandemic was key to advancing the quality of care.

## Figures and Tables

**Table 1:** Sociodemographic characteristics of the analysis sample (*N* = 141).

Sociodemographic Characteristics	*N* (%)	M (SD)
Age (Range: 23–29; Median = 26)		25.98 (1.43)
Race/ethnicity ^a^		
White	83 (59.29)	
Black or African American	36 (25.71)	
American Indian or Alaska Native	1 (0.71)	
Asian/Middle Eastern	12 (8.57)	
Hispanic/Latino	8 (5.71)	
Current Employment Status		
Full-time paid position	105 (74.47)	
Part-time paid position	15 (10.64)	
Volunteer position	1 (0.71)	
Not working	20 (14.18)	
Current Residential Status		
Living in own residence	85 (60.28)	
Living in a shared residence	46 (32.62)	
Living in the residence of a family member	10 (7.09)	
Gender ^a^		
Woman	86 (60.99)	
Man	48 (34.04)	
Gender Diverse	7 (4.96)	
Sexual Orientation ^a^		
Sexual minority young adults (SMYAs)	28 (19.86)	
Heterosexual young adults (HYAs)	113 (80.14)	

Note. Percentages may not equal 100 due to missing data. ^a^ Participants could select more than one response option.

**Table 2:** Means, standard deviations, internal reliability estimates, and bivariate correlations among continuous study variables.

	M	SD	α	1.	2.	3.	4.	5.	6.	7.	8.	9.
Age	25.98	1.43	--	1								
2. Disease-related Isolation	0.36	0.36	0.63	−0.16	1							
3. Social Network-related Isolation	0.78	0.26	0.55	−0.02	0.04	1						
4. Loneliness	1.91	0.68	0.95	−0.00	−0.02	0.04	1					
5. Family Closeness	1.56	0.73	0.94	0.01	0.08	−0.01	−0.22 *	1				
6. T1 Problematic Alcohol Use	0.00	0.94	0.81	−0.03	−0.05	0.11	−0.04	0.04	1			
7. T1 Problematic Drug Use	0.00	0.91	0.72	−0.09	−0.02	0.10	−0.01	0.04	0.96 ***	1		
8. T2 Problematic Alcohol Use	0.00	0.90	0.76	−0.18 *	−0.08	0.04	0.13	0.01	0.61 ***	0.58 ***	1	
9. T2 Problematic Drug Use	0.01	0.84	0.67	−0.23 **	−0.13	0.02	0.10	−0.00	0.58 ***	0.64 ***	0.82 ***	1

Note. * *p* < 0.05; ** *p* < 0.01; *** *p* < 0.001.

**Table 3:** Multivariate regression results: Associations between covariates and problematic substance use (model 1) and between sexual orientation and problematic substance use (model 2).

Model 1						
	T2 Problematic Alcohol Use			T2 Problematic Drug Use		
Predictors	Estimate (b)	Test Statistic (t)	*p*-Value (*p*)	Estimate (b)	Test Statistic (t)	*p*-Value (*p*)
Age	−0.12	−2.50	0.01 *	−0.11	−2.42	0.02 *
Race/Ethnicity	0.03	0.19	0.85	−0.06	−0.42	0.68
Employment Status	−0.12	−0.64	−0.52	0.14	0.78	−0.44
Residential Status	−0.02	−0.16	0.88	0.03	0.25	0.80
T1 Problematic Alcohol Use	0.81	3.14	0.002 **	−0.33	−1.32	0.19
T1 Problematic Drug Use	−0.18	−0.67	0.51	1.02	3.98	0.000 ***
**Model 2**						
	**T2 Problematic Alcohol Use**			**T2 Problematic Drug Use**		
**Predictors**	**Estimate (b)**	**Test Statistic (t)**	***p*-Value (*p*)**	**Estimate (b)**	**Test Statistic (t)**	***p*-Value (*p*)**
Age	−0.10	−2.26	0.02 *	−0.12	−2.64	0.01 *
T1 Problematic Alcohol Use	0.67	14.06	0.000 ***			
T1 Problematic Drug Use				0.71	14.51	0.000 ***
Sexual Orientation	0.15	0.95	0.34	0.03	0.19	0.85

Note. * *p* < 0.05; ** *p* < 0.01; *** *p* < 0.001.

## Data Availability

Data are available upon request to the corresponding author.
